# Self-Inflicted Urethrovesical Foreign Bodies in Children

**DOI:** 10.1155/2012/134358

**Published:** 2012-08-09

**Authors:** Canan Ceran, Sema Uguralp

**Affiliations:** Department of Pediatric Surgery, Faculty of Medicine, İnönü University, 44280 Malatya, Turkey

## Abstract

We present two cases of self-inflicted urethrovesical foreign body in children. Case 1 was a 6-year-old girl admitted with a history of self-introduction of a pin. The X-ray revealed the pin as 3.5 cm in length and in the bladder. The foreign body was removed endoscopically. Case 2 was a 13-year-old boy with a self-introduced packing needle, 13 cm in length, partially in the urethra. The end and the tip of the needle passed through the urethra to the surrounding tissues. Foreign body removed via a little skin incision with endoscopic guidance. Foreign bodies are rarely found in the lower urinary tract of children. Definitive treatment is usually the endoscopic removal; however, sometimes surgical intervention may require.

## 1. Introduction

Self-introduction of the foreign body (FB) into the urethra and bladder in children has been rarely reported in the literature [1]. FBs were inserted or applied to the urethra for autoerotic, psychiatric, therapeutic purposes, or no definite reasons by the patient [2]. Majority of such cases are adult men. FBs are rarely found in the bladder of children.

In this paper, we present two cases of self-inflicted foreign bodies through their urethra and review the literature.

## 2. Case 1

**Figure 1:**
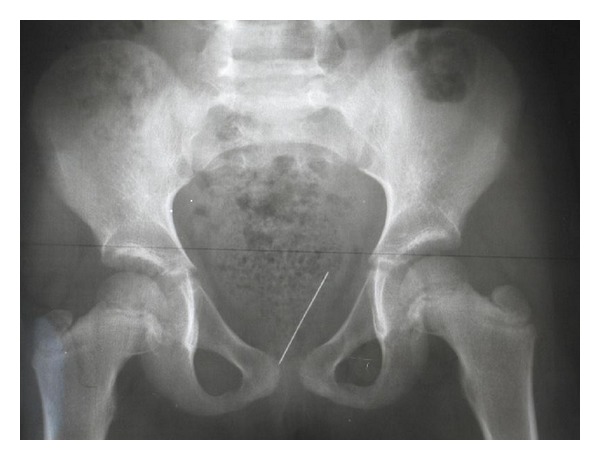
Posteroanterior pelvic radiogram of case  1. There is a linear, radio-opaque FB in the pelvis.

A 6-year-old girl was admitted to the emergency service with a history of self-introduction of a pin into her genital area approximately 3 hours after the event. The patient was asymptomatic, and the physical examination of the patient revealed normal findings with no sign of trauma at the external genitalia. Urinanalysis was normal. Posteroanterior and lateral pelvic radiogram showed a pin lies in the pelvis (Figure 1).

**Figure 2:**
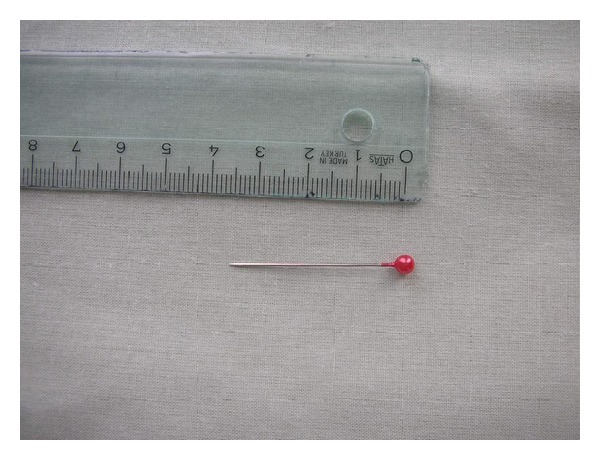
The pin which was removed from bladder has one sharp end and one plastic bead.

Cystoscopy confirmed that a pin lies within the bladder. The pin has one sharp end and one plastic bead (Figure 2). The attempt of grasping to the pin with forceps in a suitable position for extraction was unsuccessful. Telescope was moved to backward, and the sharp end of the pin was taken into the cystoscope's sheath (9 Fr). Then, the telescope was moved forward and the pin was caught between the sheath and the telescope. The pin was removed via transurethral route with cystoscope. The patient was discharged with psychiatric referral.

## 3. Case 2

**Figure 3:**
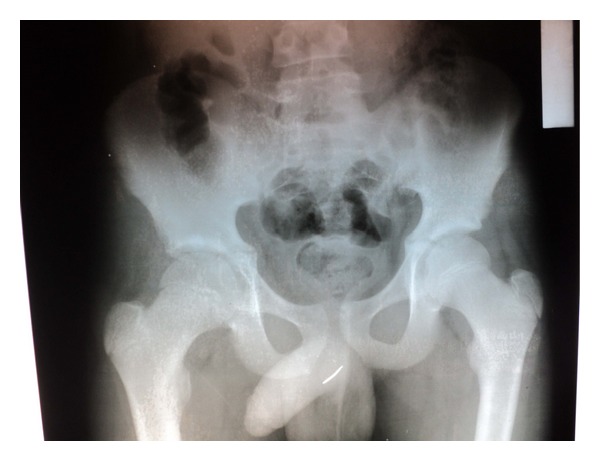
Posteroanterior pelvic radiogram of case  2 showed that there is a linear radio-opaque shadow in the region of bulbar urethra. In this graph the foreign body seems about 3 to 4 cm because of its direction.

**Figure 4:**
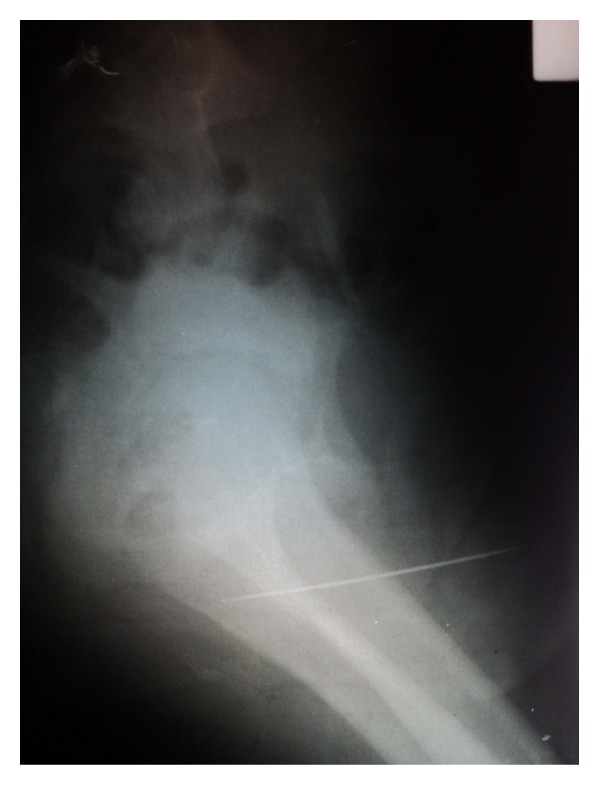
Lateral pelvic radiogram of case  2 revealed that FB was about 13 cm in length.

Thirteen-year-old boy was admitted to the pediatric surgery department with a history of self-insertion of a packing needle same day. It was associated with perineal pain and dysuria. Posteroanterior pelvic radiogram revealed a fine, linear radio-opaque shadow in the region of bulbar urethra. In this graph, foreign body seems to be about 3 to 4 cm because of its direction (Figure 3). Lateral X-ray graph revealed that FB was about 13 cm long (Figure 4).

Urethroscopy with 9 Fr cystoscope revealed a metallic FB located at posterior urethra. The pinpoint and the end point of the needle were out of the urethra, and mid portion is in the urethra. The pinpoint of the needle was palpated just posterior to the scrotum. In this region, skin incised and the needle was grasped then extracted. We avoided from urethrotomy. The patient was postoperatively managed 7 days with urethral catheter. Then, foley catheter was removed and the patient discharged. The patient diagnosed as obsessive and compulsive after psychiatric evaluation and medical treatment was started.

## 4. Discussion

FBs in the lower urinary tract may result from self-insertion, migration from adjacent sites, that may be iatrogenic or traumatic. The reasons for introduction of objects into the urinary tract could be psychiatric, accidental, sexual stimulation, curiosity especially among children, or therapeutic in cases of stricture [3]. 

Self-introduction of the FBs is rarely seen in children [4]. Generally, they are firstly observed at the start of puberty as in case  2 [1, 5]. As seen in case  1, self-introduction before puberty is very rare. Both of our patients inserted needle, an unusual FB for such a patient. The types of FBs include plastic caps, hooked wire, paper clips, metal objects, glass rods, shells, light bulbs, and so forth. Multiple urethral FBs also have been described [3].

Urethrovesical FBs rarely present in a clear clinic circumstances, rather, there are often suspicious stories of trauma or urinary complaints. Most patients are too ashamed to admit that they had inserted or applied any object and usually presented when a complication had occurred from the FB [2, 5]. Fortunately, our patients probably due to the innocence of their age group told their parents that they put a pin in their genital area and then pin was disappeared so foreign body diagnosed immediately.

Patients usually present with dysuria. Other presenting compliant includes difficulty in voiding, hematuria, pain, swelling of genitalia, extravasations of urine, abscess formation, and purulent discharge [4, 5].

Evaluation of the patient focuses on ascertaining-detailed information about the foreign FB—particularly composition, size, and shape—to establish the risk of trauma to the urethra or the bladder perforation. A plain abdominal X-ray followed by cystoscopy usually suffices for the diagnosis of the presence and the location of the FB. Most urethrovesical FBs are visible on the plain radiographs. Ultrasound has also been employed [6]. In some cases for the diagnosis, it is also required to have a computed tomography [7]. Occasionally, cystourethrography is needed.

Definitive treatment is removal of FB which is usually performed via cystoscopy but may require open surgery [5]. Even in infancy it is possible to extract FBs by transurethral approach. Endoscopy is the least invasive technique for retrieval. Objects too large for transport preclude its use as urethral trauma can occur. Ingenious modifications of conventional instruments have been described to tackle difficult FBs. In case  1, we had difficulties to grasp the pin, and we removed it by novel manipulation of the instruments. Some authors advocate that percutaneous suprapubic retrieval under direct visualization via cystoscopy is the technique of choice in children [8]. Open exploration is the most invasive but also the most successful technique. In case  2, tip of the needle was found in the subcutaneous tissue via a small skin incision and the needle was grasped than extracted. Generally, patients do well following removal.

Septic and mechanic complications including urinary tract infection, bladder perforation, calcification, bleeding, sepsis, and outflow obstruction may occur due to the urethrovesical FBs. Sivaloganathan had reported a patient who presented quite late and died because of sepsis resulting from a vesical FB [9]. If treatment is delayed, a chronic condition develops in these cases and repeating infections such as urinary retention, squamous cell carcinoma, urethral stenosis, calcification of FB, and migration of FB and stone can occur [3, 10].

Various organisms have been isolated from the urine. Urine culture could have been sterile as in our patients [4]. Bulow recommended that urinary tract infections which nearly always accompany the presence of FBs in the urinary tract should be treated with antimicrobials [11]. We did not use antibiotic because urine culture of the patient was sterile in case  1. In case  2 we used antibiotics until urethral catheter removal.

Psychiatric evaluation has been advised in all cases of self-introduction of FB, although this has not been universally agreed upon. Prepubertal children usually have introduced objects out of normal childhood curiosity as in case  1 and case  2 were obsessive and compulsive.

There are only a few case reports of self-insertion of foreign bodies in the bladder among children. Moreover, the insertion of pin is unusual. The diagnosis and management of urethrovesical FBs require expertise. Endoscopic and minimal invasive techniques should be used.
